# Amaranth Genomic Resource Database: an integrated database resource of Amaranth genes and genomics

**DOI:** 10.3389/fpls.2023.1203855

**Published:** 2023-06-28

**Authors:** Akshay Singh, Ajay Kumar Mahato, Avantika Maurya, S. Rajkumar, A. K. Singh, Rakesh Bhardwaj, S. K. Kaushik, Sandeep Kumar, Veena Gupta, Kuldeep Singh, Rakesh Singh

**Affiliations:** ^1^ Division of Genomic Resources, ICAR-National Bureau of Plant Genetic Resources, New Delhi, India; ^2^ The Centre for DNA Fingerprinting and Diagnostics, Hyderabad, India; ^3^ Division of Germplasm Evaluation, ICAR- National Bureau of Plant Genetic Resources, New Delhi, India; ^4^ Division of Germplasm Conservation, ICAR- National Bureau of Plant Genetic Resources, New Delhi, India; ^5^ International Crop Research Institute for the Semi-Arid Tropics, Hyderabad, India

**Keywords:** Amaranth Genomic Resource Database, SSRs, SNPs, TFs, miRNAs, Transporters

## Abstract

Amaranth (*Amaranthus* L.) is native to Mexico and North America, where it was cultivated thousands of years ago, but now amaranth is grown worldwide. Amaranth is one of the most promising food crops with high nutritional value and belongs to the family Amaranthaceae. The high-quality genome assembly of cultivated amaranth species (*A. hypochondriacus*, *A. cruentus*) and wild/weedy species (*A. tuberculatus*, *A. hybridus*, and *A. palmeri*) has already been reported; therefore, we developed an Amaranth Genomic Resource Database (AGRDB) to provide access to all the genomic information such as genes, SSRs, SNPs, TFs, miRNAs, and transporters in one place. The AGRDB database contains functionally annotated gene information with their sequence details, genic as well as genomic SSRs with their three sets of primers, transcription factors classified into different families with their sequence information and annotation details, putative miRNAs with their family, sequences, and targeted gene details, transporter genes with their superfamily, trans-membrane domain details, and details of genic as well as nongenic SNPs with 3′ and 5′ flanking sequence information of five amaranth species. A database search can be performed using the gene ID, sequence ID, sequence motif, motif repeat, family name, annotation keyword, scaffold or chromosome numbers, etc. This resource also includes some useful tools, including JBrowse for the visualization of genes, SSRs, SNPs, and TFs on the respective amaranth genomes and BLAST search to perform a BLAST search of the user’s query sequence against the amaranth genome as well as protein sequences. The AGRDB database will serve as a potential platform for genetic improvement and characterization of this futuristic crop. The AGRDB database will be accessible via the link: http://www.nbpgr.ernet.in:8080/AmaranthGRD/.

## Introduction

Amaranth (*Amaranthus* L.) is a historically important, ancient paleopolyploid, C4 dicotyledonous plant species that belongs to the family Amaranthaceae, subfamily Amaranthoideae (Clouse et al., 2016; Thapa and Blair, 2018). This species is getting renewed interest as a nutri-cereal from researchers and consumers alike, especially health-conscious populations suffering from modern-world lifestyle diseases like diabetes and hypertension (Ruth et al., 2021). Amaranth is a fast-growing crop and one of the cheapest dark green vegetables on the tropical market due to its low production costs. It is often referred to as the poor man’s vegetable (Rastogi and Shukla, 2013). It is considered a “superfood” or millennium crop now-a-days because of its high nutraceutical value, such as high-quality proteins, unsaturated oils, dietary fibers, flavonoids, vitamins, and essential minerals (Soriano-García et al., 2018). Amaranth grains are a rich source of minerals such as calcium, magnesium, and copper, as well as sodium, iron, phosphorus, and zinc. It also contains vitamins such as thiamine, riboflavin, ascorbic acid, and niacin (Palombini et al., 2013; Joshi et al., 2018). Regular consumption of amaranth seed or seed oil provides a significant amount of vitamin E and squalene, which benefit people suffering from hypertension or cardiovascular disease by lowering blood pressure and cholesterol levels and improving antioxidant status (Martirosyan et al., 2007). The phytochemical examination of dried amaranth grains revealed the presence of good-quality alkaloids, phenolics, flavonoids, and saponins (Barba de la Rosa et al., 2009). Considering the adverse effects of changing climatic conditions, amaranth is a promising agricultural crop with the ability to withstand negative growing conditions, including resistance to drought, heat, salinity, pests, and adaptability to environments (Alemayehu et al., 2014; Aderibigbe et al., 2020).

However, despite the nutritional and agricultural importance of this crop, it is still one of the most underexploited crops globally, including in India. Genomic information is essential for the effective genetic improvement of any crop. In recent years, due to the rapid advancement of sequencing technologies, high-quality genome sequences of several amaranth species have been published (Sunil et al., 2014; Lightfoot et al., 2017; Montgomery et al., 2020; Ma et al., 2021; Wang et al., 2022). These developments in sequencing led to the generation of a large number of transcriptome and population DNA re-sequencing data, including mining key genes related to important agronomic traits, to explore gene function, genetic diversity, and evolutionary analysis not only in amaranth but also in other *Amaranthaceae* members (Délano-Frier et al., 2011; Hoshikawa et al., 2023; Vats et al., 2023). To date, there exists a database tool for amaranth named AmaranthGDB (Gonçalves-Dias and Stetter, 2021), which provides an amaranth population genetics genome browser (PopAmaranth). This browser facilitates browsing through three different categories of summary statistics, namely genetic diversity, population differentiation, selection signals, gene annotation, and variant calls, along with the amaranth genome (Gonçalves-Dias and Stetter, 2021). However, this database tool does not provide any information on related genes, SSRs, TFs, miRNAs, transporters, or their sequences, as well as functional annotation information, so users need to extract this information themselves. Accordingly, a database is required that makes it possible to efficiently integrate all gene and genomics-related information for amaranth species, analyze multi-omics data, and provide a platform for researchers to quickly access and utilize these resources. Several genomic resources are already available for various plant and crop species, such as the Tomato Genomic Resources Database (TGRD) for tomato (*Solanum lycopersicum*) (Suresh B. V. et al., 2014), the Date Palm Genomic Resource Database (DRDB) for date palm species (He et al., 2017), the TeaPGDB for tea species (Lei et al., 2021), and the Chickpea genomic web resource for *Cicer arietinum* (Misra et al., 2014).

In the present study, in addition to the existing databases, a dedicated database containing complete information on genes with their functional annotation, molecular markers (SSRs and SNPs), TFs, putative miRNAs with their target genes, and transporter genes of the genus *Amaranth* has been developed. The availability of such genomic resources and important tools as Gene Expression, BLAST Search, and JBrowse has made it possible to examine important and complex traits in the breeding process more effectively. AGRDB will be updated regularly in accordance with the availability of the sequence of new amaranth species and improved versions of the existing amaranth species, as well as the incorporation of user suggestions for the improvement of our AGRDB database. This database will be handy to researchers for selecting desired molecular tools to assist in breeding programs for genetic improvement of this nutritionally important, climate-resilient crop in the future.

## Materials and methods

### Sequence data retrieval

For the development of the AGRDB database, complete genome sequence data for *A. hypochondriacus* from Phytozome V13 (https://phytozome.jgi.doe.gov/Ahypochondriacus_er), *A. tuberculatus*, *A. hybridus*, and *A. palmeri* from a Comparative Genomics Platform CoGe: (https://genomevolution.org/coge/; Genome ID 54057, 57429, 56750), and *A. cruentus* was downloaded from NCBI database (https://www.ncbi.nlm.nih.gov/genome/109717?genome_assembly_id=1765032). The assembled genomes of *A. hypochondriacus*, *A. tuberculatus*, *A. hybridus*, and *A. palmeri* were scaffold-wise, and *A. cruentus* as 17 pseudomolecules, including chromosomes 2A and 2B (**Table 1**). The quality of the assembled genome sequences used in this study was checked using Long Terminal Repeat (LTR) Assembly Index (LAI) method (Mokhtar et al., 2023). The genomes of *A.hypochondriacus* (GCA_00753965.2; LAI score: 12.81), *A. tuberculatus* (Genome ID: 54057; LAI score: 6.72), *A. hybridus* (Genome ID: 57429; LAI score: 9.56), *A. palmeri* (GCA_025765695.1; LAI score: 4.99), and *A. cruentus* (GCA_019425755.1; LAI score: 14.42), respectively.

**Table 1:** The genome sequence data of different amaranth species used in this study.

Species	Assembly version	Assembly level	Genome size (Mb)	GC (%)
*A. hypochondriacus*	Ver.2.0 (Phytozome)	Scaffold	403.89	32.65%
*A. tuberculatus*	Ver.2.0 (CoGe)	Scaffold	688.98	33.14%
*A. hybridus*	Ver.1.0 (CoGe)	Scaffold	411.83	32.30%
*A. palmeri*	Ver.1.2 (CoGe)	Scaffold	411.92	33.18%
*A. cruentus*	Ver.1.0 (NCBI)	Chromosome	365.20	33.03%

### SSR identification and primer generation

The whole genome sequences of *A. hypochondriacus*, *A. tuberculatus*, *A. hybridus*, *A. palmeri*, and *A. cruentus* were used as input to the microsatellite identification tool Krait v1.3.3 (https://github.com/lmdu/krait) (Du et al., 2017) to identify SSR markers using the parameters: mono-nucleotide repeats were not considered; the minimum number of repeats allowed for the di-nucleotide were six repeats; and for tri-nucleotide to hexanucleotide, five repeats (Paliwal et al., 2016; Singh et al., 2022). The maximum difference between the two SSRs is 100 bp. Primer pairs were generated from the 250-bp flanking region of each identified SSRs using Primer3 software (Untergasser et al., 2012) with the parameters: primer length = 20–25 bp, PCR product size = 100–300 bp, with an optimum of 180 bp; annealing temperature = 65°C; GC content of (40%–60%) with optimal value 50%. The genomic coordinates (exons, introns, 3′UTRs, 5′UTRs, and intergenic region) were extracted from the gff file and genome using bed tool utilities (Quinlan and Hall, 2010).

### Identification of SNPs and TFs

High-quality SNP mining involves multiple steps using various bioinformatics tools. The BioProject numbers PRJNA290898, PRJNA432348, and PRJNA626536 have been used for SNP mining, and the details of SRA accessions are provided in **Supplementary Table S1**. After a quality check by the FASTQC toolkit, trimming of raw reads was performed using Trimmomatic (Bolger et al., 2014). The resulting high-quality reads were mapped to the corresponding amaranth reference genomes using the BWA aligner with default parameters (Li and Durbin, 2009). The read-mapped alignment file is in SAM format. SAMTools was used to convert SAM to BAM file format, and the BAM file was then shortened by removing duplicate reads (Li et al., 2009). SNP calling and filtering were performed using SAMTools, VarScan, and bcftools. The criteria for filtering the SNPs were Q score ≥ 30, and X-coverage ≥ 30×. We used an in-house Perl script to extract the final high-quality SNPs and their location within the gene region, as well as 3′ and 5′ flanking sequences. The identification of candidate genes for transcription factor families in *A. hypochondriacus*, *A. tuberculatus*, *A. hybridus*, *A. palmeri*, and *A. cruentus* was performed by BLASTX similarity search of the protein-coding genes of the respective amaranth species against available peptide sequence of plant transcription factor database (PlantTFDB v4.0) (Jin et al., 2016), with the parameters bit score >100 and *e*-value 1 × 10^−5^ (Singh et al., 2017). All the identified TFs were categorized into 57 different families, and their functional annotation was performed using InterProScan 5 (Jones et al., 2014).

### Identification of miRNAs and transporters

*In silico* prediction of the potential miRNAs was performed using BLASTN search of a set of mature miRNA sequences against the *A. hypochondriacus*, *A. tuberculatus*, *A. hybridus*, *A. palmeri*, and *A. cruentus* genome sequences. A total of 7,043 nonredundant mature miRNA sequences retrieved from the PMRD-plant microRNA database (Zhang et al., 2009) were used as the database in BLASTN search with the following parameters: (i) word match size, 7; (ii) length of mature miRNA sequence, ≥ 18 nucleotides without gap; (iii) mismatch range, 0–2; and (iv) *e*-value, 0.1. The sequences with less than two nucleotide mismatches were allowed, and 200 nucleotides upstream and downstream from the matched region were extracted for each miRNA (pre-miRNA sequences/possible miRNA precursors). Furthermore, sequences coding for proteins were eliminated, whereas the noncoding regions were considered for secondary structure prediction and validation. The MFold web server (http://www.unafold.org/) was used to generate and evaluate the potential secondary structures of pre-miRNAs. Screening of pre-miRNAs with the presence of proper stem-loop hairpin structure, and minimum free energy (MFE) values were calculated for each secondary structure and filtered for the secondary structure of pre-miRNAs with the lowest MFE values (Sharma et al., 2019). The pipeline for *in silico* miRNA prediction used in this study has been presented in **Supplementary Figure S1**. Target identification of the predicted candidate miRNAs was done using the online tool psRNAtarget (http://plantgrn.noble.org/psRNATarget/) (Dai et al., 2018), using the option “Submit small RNAs and target” with default parameters. To identify transporter genes in *A. hypochondriacus*, *A. tuberculatus*, *A. hybridus*, *A. palmeri*, and *A. cruentus*, the protein sequences of respective amaranth species were subjected to BLASTP search against the transporter classification database (TCDB; http://www.tcdb.org/) (Saier et al., 2009), with *e*-value and bit score cutoffs of 10^−5^ and 100, respectively. The presence of the transmembrane domain was analyzed using TMHMM server v2.0 (Sonnhammer et al., 1998). The transporter genes with > 2 transmembrane (TM) domains were filtered out, and the classification of such genes in different subfamilies was done using the TCDB database.

### Database architecture

**Figure 1:**
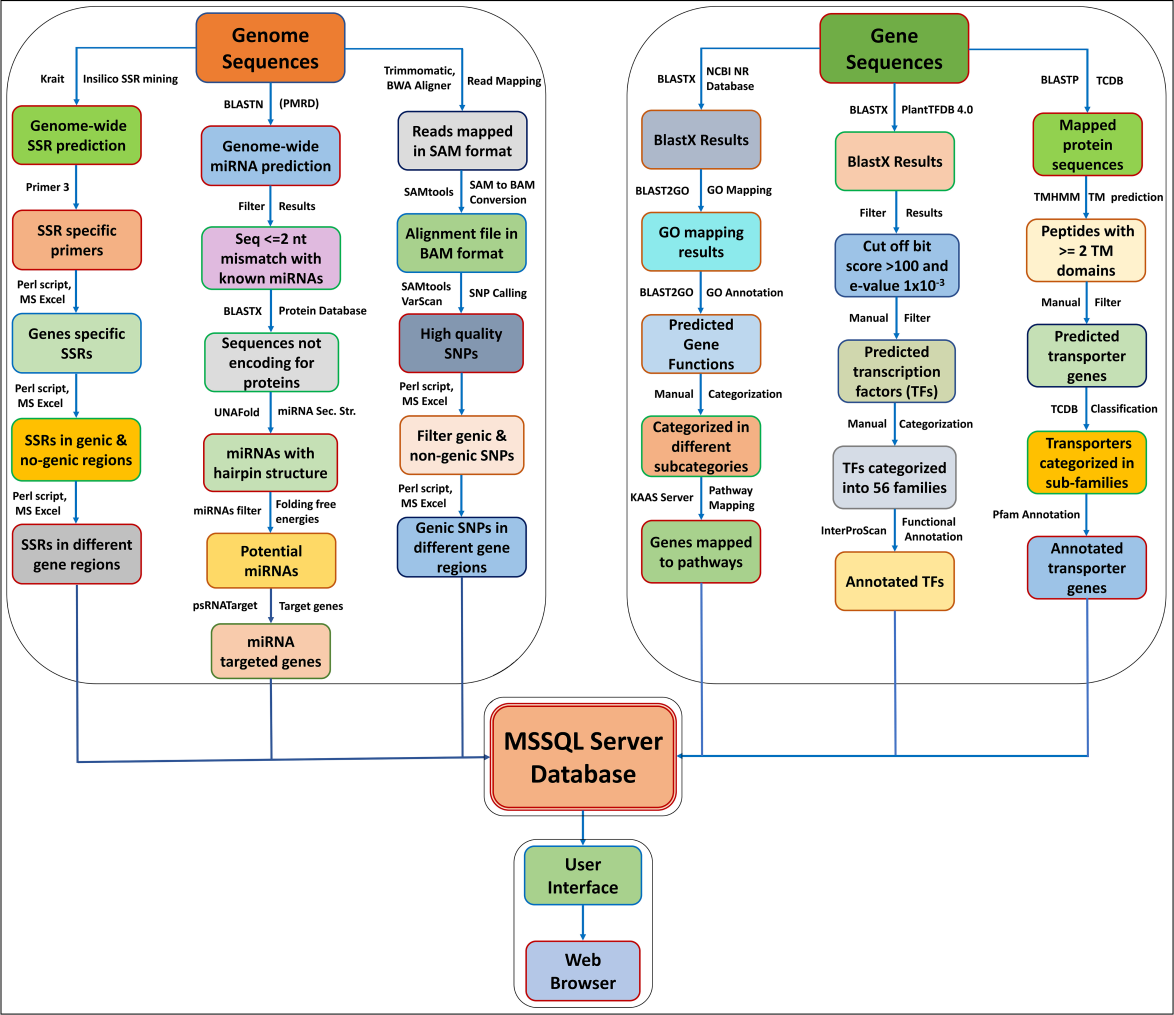
A schematic workflow of the AGRDB database showing the data generation pipeline shows the steps and software tools used to generate the useful data present in the AGRDB database.

The AGRDB database is a relational and interactive online database based on a “three-tier schema architecture” with client, server, and database tiers. The interactive, user-friendly interface of the AGRDB database has been designed using server-side programming languages such as ASP.NET, JavaScript, and Microsoft SQL Server to store large database tables. The AGRDB database contains step-by-step graphical tutorials to help users make better use of the database and a local NCBI BLAST server for nucleotide and protein sequence similarity searches. A schematic workflow of the AGRDB database showing the data generation pipeline has been presented in **Figure 1**, and the database schema is represented in **Supplementary Figure S2**.

## Results

### Overview of the database

The AGRDB database comprises detailed genomic information on five amaranth species, essential for the genetic improvement of the amaranth species. Data have been stored in the MSSQL Server Database in separate tables named, (i) *Gene_details* contains general information about the genes, such as gene ID, scaffold/chromosome number, genomic position, function, and sequence; (ii) *SSR_details* contains general information about SSRs, such as SSR type, SSR motif sequence, motif repeat, SSR length, and their three pairs of primer information; (iii) *SNP_details* stores general information about SNPs present in genic regions (exon, intron, 3′UTR, 5′UTR), as well as nongenic regions with 100 bp flanking sequences of both 3′ and 5′ regions; (iv) *TF_details* contains details about TFs and their annotation information, (v) *miRNA_details* stores general information about miRNAs, targeted genes, and their annotation information, and (vi) *Transporter_details* contains general information about transporter genes mentioned in **Table 2**.

**Table 2:** The number of entries stored in the AGRDB database tables: Gene details, SSR_details, SNP_details, TF_details, miRNA_details, and Transporter_details.

Species	Gene_details	SSR_details	SNP_details	TF_details	miRNA_details	Transporter_details
***A. hypochondriacus***	23,879	205,567	958,646	7,163	42	2,760
***A. tuberculatus***	30,771	165,246	1,570,771	8,685	36	2,905
***A. hybridus***	23,820	102,492	991,082	6,655	39	2,642
***A. palmeri***	48,625	106,586	760,209	19,481	38	3,402
***A. cruentus***	43,382	78,445	1,321,409	17,665	36	2,760
**Total**	**170,477**	**658,336**	**5,602,117**	**59,649**	**191**	**14,469**

### Database web interface

**Figure 2:**
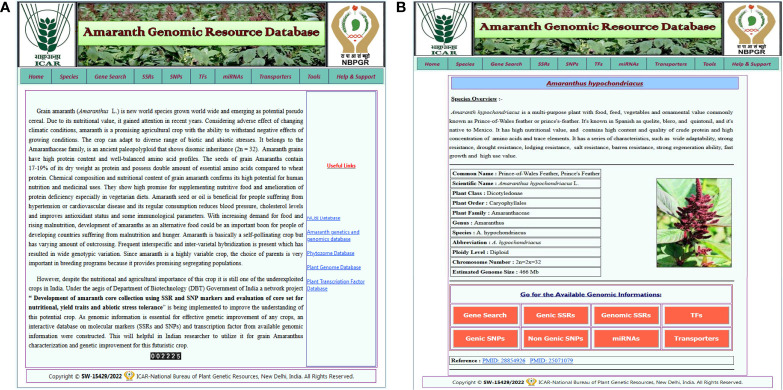
The graphical user interface of the AGRDB database. **(A)** The home page displays the various search tabs available in the AGRDB database. **(B)** The species search page contains a brief description of the amaranth species as well as links to get the genomic information for that species.

The AGRDB database comprises a user-friendly web interface and multiple search options for systematic data retrieval. The database has been stratified into 10 different tabs, including home, species, gene search, SSRs, SNPs, TFs, miRNAs, transporters, tools, and help and support (**Figure 2A**). The “home” page provides an overview of the database and about nutritional as well as medicinal importance of grain amaranth. The “species” tab is redirected to the separate amaranth species page that gives a short description of selected species and links to access the genomic information of that species (**Figure 2B**). The “SSRs,” “SNPs,” “TFs,” “miRNAs,” and “transporters” pages contain different search functions such as unique ID, scaffold or chromosome number, motif sequence, annotation keywords, etc. By using these search criteria, users can retrieve the information as per their requirements. Under the “tools” tab, gene expression, the local BLAST server, and JBrowse have been configured. The “help and support” tab contains a downloads page, a tutorial to access the AGRDB database more efficiently, and contact information for the users to send their queries or suggestions if they face any difficulty in accessing the database.

### Key features of the AGRDB database

The AGRDB database accommodates various search menus under different sections for data retrieval and annotation information.

#### Gene and SSR search

**Figure 3:**
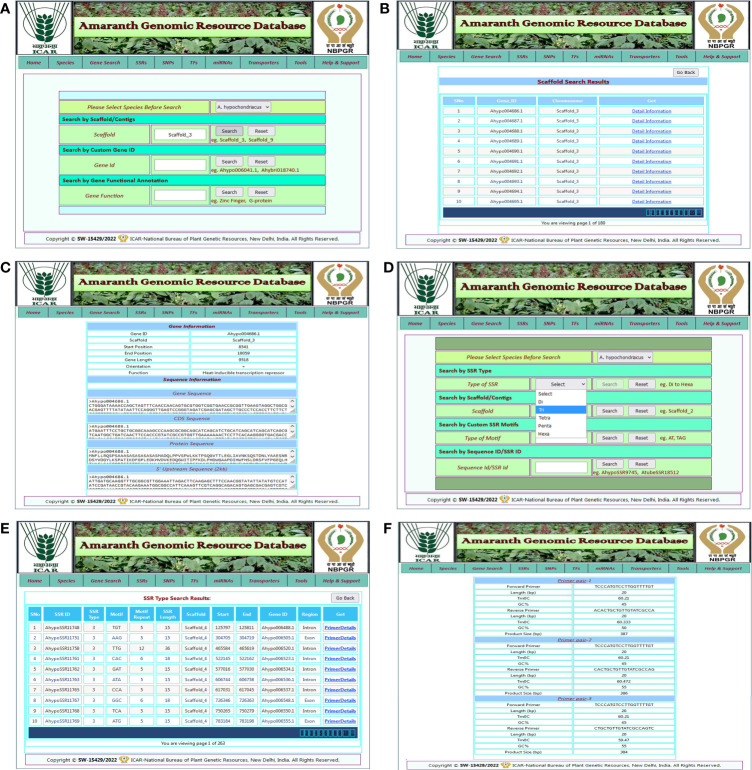
Example of the gene search, SSR search features, and generated results from the AGRDB database. **(A)** The different search modules for species-specific gene search; the search was performed with the scaffold search option using scaffold_3. **(B)** The list of genes present in scaffold_3 of selected species. **(C)** The detailed information of selected genes of interest. **(D)** The different search modules for species-specific SSR search and the search was performed with the SSR type search option, trinucleotide. **(E)** A result page showing the list of trinucleotide SSR details in selected species and a link for primer details for that SSR marker. **(F)** A result page displaying three pairs of primer sequences for the corresponding SSR marker.

The gene search page enables the user to get information about protein-coding genes, gene IDs, scaffold numbers, genomic positions, functional annotations, and sequences of all five amaranth species. The gene search utility facilitates the retrieval of amaranth gene information based on gene ID, scaffold number, and gene functional annotation keyword (**Figure 3A**). Users can select the species name from the dropdown menu and enter any of the search criteria, such as scaffold number, gene ID, or gene functional annotation, and hit the search button. A new result page will then open that contains columns of gene ID, scaffold number, and a link to get detailed information about the selected gene (**Figure 3B**). When users click on the “detail information” link, a separate page will open that contains detailed information about the gene, such as its gene ID, scaffold number, genomic position, gene length, orientation, functional annotation, gene sequence, CDS sequence, peptide sequence, and 2 kb upstream sequence (**Figure 3C**).

The SSR search page enables the analysis of genic as well as genomic SSRs detected across five amaranth genome species. Both the genic and genomic SSR search tab contain a form where users first select the species name from the dropdown list, then provide any one of the search criteria given, such as SSR type, scaffold number, motif sequence type, or SSR ID and hit the search button (**Figure 3D**). After the search, the user is redirected to a new result page that contains full SSR details such as SSR ID, SSR type, SSR motif, motif repeat type, SSR length, scaffold number, gene ID, and region, along with a link to get primer details (**Figure 3E**). By clicking on the link “primer details,” the user goes to a new result page containing full details of three pairs of primers for selected SSR, such as primer sequence, lengths, Tm°C, GC%, and product size of the SSR primer (**Figure 3F**).

#### TF search

**Figure 4:**
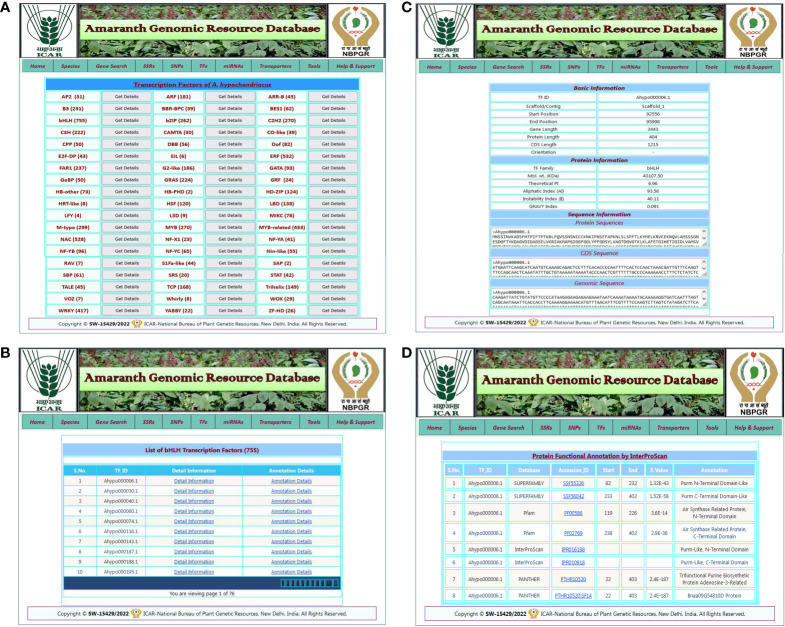
An example of the TF search and its associated result pages. **(A)** A list of 57 TF categories predicted in selected amaranth species *A. hypochondriacus*. **(B)** The result page shows a list of TFs predicted in the selected TF category with the links for detailed information and annotation details of that transcription factor. **(C)** The detailed information for the selected transcription factor. **(D)** A result page containing functional annotation details of selected transcription factors predicted from the InterProScan database.

The TF search allows users to access detailed information about various transcription factor families present in five amaranth species. The TF search tab redirected to the TF page of the respective amaranth species, which contains a list of predicted TFs in that amaranth species categorized into 57 TF categories with a “get details” link for each TF category (**Figure 4A**). By clicking on the get details link, the user is redirected to a new result page that contains a list of TFs present in that TF category and two links: detail information and annotation details (**Figure 4B**). By clicking on the “detail information” link, the user is redirected to the next result page that contains basic information (TF ID, scaffold, start, and end positions, gene length, CDS length, protein length, and orientation), protein information (TF family, molecular weight, isoelectric point, aliphatic index, instability index, and GRAVY index), and sequence information (protein sequence, CDS sequence, and genomic sequence) of that transcription factor (**Figure 4C**). When the user clicks on the “annotation details” link, they are redirected to a new result page that contains functional annotation details of the respective TF from the InterProScan database (**Figure 4D**).

#### SNP, miRNA, and transporter search

**Figure 5:**
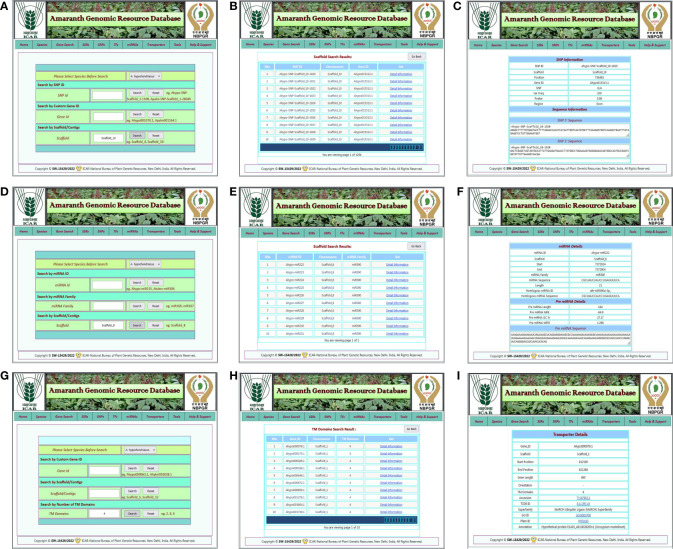
An example of SNP, miRNA, transporter searches, and result pages. **(A)** The various search modules for species-specific SNP search and the search were performed with the scaffold search option using scaffold_10. **(B)** The list of SNPs present in scaffold_10 of selected species, with a link to detailed information for corresponding SNPs. **(C)** The result page shows detailed information about selected SNPs. **(D)** The different search functions for species-specific miRNA searches and the search were performed with the scaffold search option using scaffold_8. **(E)** A result page showing the list of miRNAs identified in scaffold_8 of selected amaranth species with miRNA family details and a link for detailed information about that miRNA. **(F)** The next page displays the details of selected miRNAs, such as miRNA details, pre-miRNA details, and pre-miRNA sequence information. **(G)** The various search modules for the species-specific transporter search, with the TM domain search option and TM number = 4. **(H)** Result page displaying the list of transporters that containing four TM domains found in selected amaranth species, along with a link to detailed information about the selected transporter protein. **(I)** The result page shows detailed information about the selected transporter protein.

Various search options have been incorporated into the AGRDB database to facilitate the search for SNP markers. The SNP search page provides users with genic and nongenic SNPs identified in three amaranth species. Both genic and nongenic SNP search tabs contain a web form where the user first selects the species name of their interest from the dropdown list and select another search criterion from the SNP ID, gene ID, and scaffold number, then hits the search button (**Figure 5A**). After performing a search, users are redirected to a new result page that contains five columns with the name S.No., SNP ID, scaffold number, gene ID, and a link to get detailed information about the respective SNP (**Figure 5B**). By clicking on the “detail information” link, the user is redirected to a new result page that contains SNP information (SNP ID, scaffold number, position, gene ID, SNP, var. freq, *p*-value, region) and sequence information (100 bp of 5′ and 3′ sequences) of selected SNPs (**Figure 5C**).

The miRNA search allows users to identify the target genes of a specific miRNA and its position in the amaranth genome. Searching for miRNAs can be done using search modules such as search by miRNA ID, search by miRNA family, and search by scaffold number by first selecting the species of interest and then clicking the search button (**Figure 5D**). Following the search, the users are directed to a new result page that includes a list of miRNAs along with miRNA family and scaffold number, as well as a link to obtain more information about the selected miRNA (**Figure 5E**). By clicking on the “detail information” link, users are redirected to the next result page that contains miRNA details (miRNA ID, scaffold number, start, and end position, miRNA family, miRNA sequence, length, and sequence as well as the ID of homologous miRNA), pre-miRNA details (pre-miRNA length, MFE, GC%, MFEI), and pre-miRNA sequence information (**Figure 5F**).

The transporter search section of the AGRDB database provides access to the transporter proteins that have been identified in five amaranth species. It can be searched using gene ID, scaffold number, and the number of TM domains by first selecting the species name (**Figure 5G**). After searching, the users are redirected to a new result page that contains a list of transporter proteins with scaffold number, number of TM domains, and a link for detailed information on that transporter protein (**Figure 5H**). The detail information link redirected to a new result page that contains details of selected transporter proteins such as gene ID, scaffold number, start and end positions, gene length, number of TM domains, tcdb accession, tcdb ID, superfamily name, go ID, Pfam ID, and function annotation details (**Figure 5I**).

#### Tools (gene expression)

The gene expression page provides a search function for genes with annotated RPKM values. Users can find this function under the tools menu tab. The gene expression results are presented as a line bar chart drawn by Ajax BarChart to display RPKM values at different experimental stages of the amaranth (**Supplementary Figure S3**). The query results support online browsing and downloading to facilitate researchers conducting in-depth analysis.

#### BLAST search and JBrowse

BLAST search provides a user-friendly BLAST tool for sequence alignment using SequenceServer (Priyam et al., 2019). Nucleotide and amino acid sequence similarity searches can be performed through a user-friendly input–output interface. We provide genomic sequences and protein sequences as databases (**Supplementary Figure S4**). Users can search the nucleotide sequence and the protein sequence databases by querying sequences in BLASTN or BLASTP search, respectively.

The genome browser is an important tool for the visualization of high-throughput sequencing data. JBrowse (Buels et al., 2016) is a genome browser based on HTML5 and JavaScript that contains a fully dynamic AJAX interface. We integrated genome, gene, SSR, and TF information for all five amaranth species and SNP information for *A. hypochondriacus*, *A. palmeri*, and *A. tuberculatus* due to the unavailability of RNASeq data for other species. On the left-hand side of the genome browser, the “Available Tracks” option provides all displayable file options. After choosing which files to display, the information will appear in a window located on the right-hand side (**Supplementary Figure S5**). Clicking on the different parts of the sequences will display detailed data information and allows users to browse gene sequences, chromosome number, SSR details, TF details, etc.

### Statistical overview of the data

**Figure 6:**
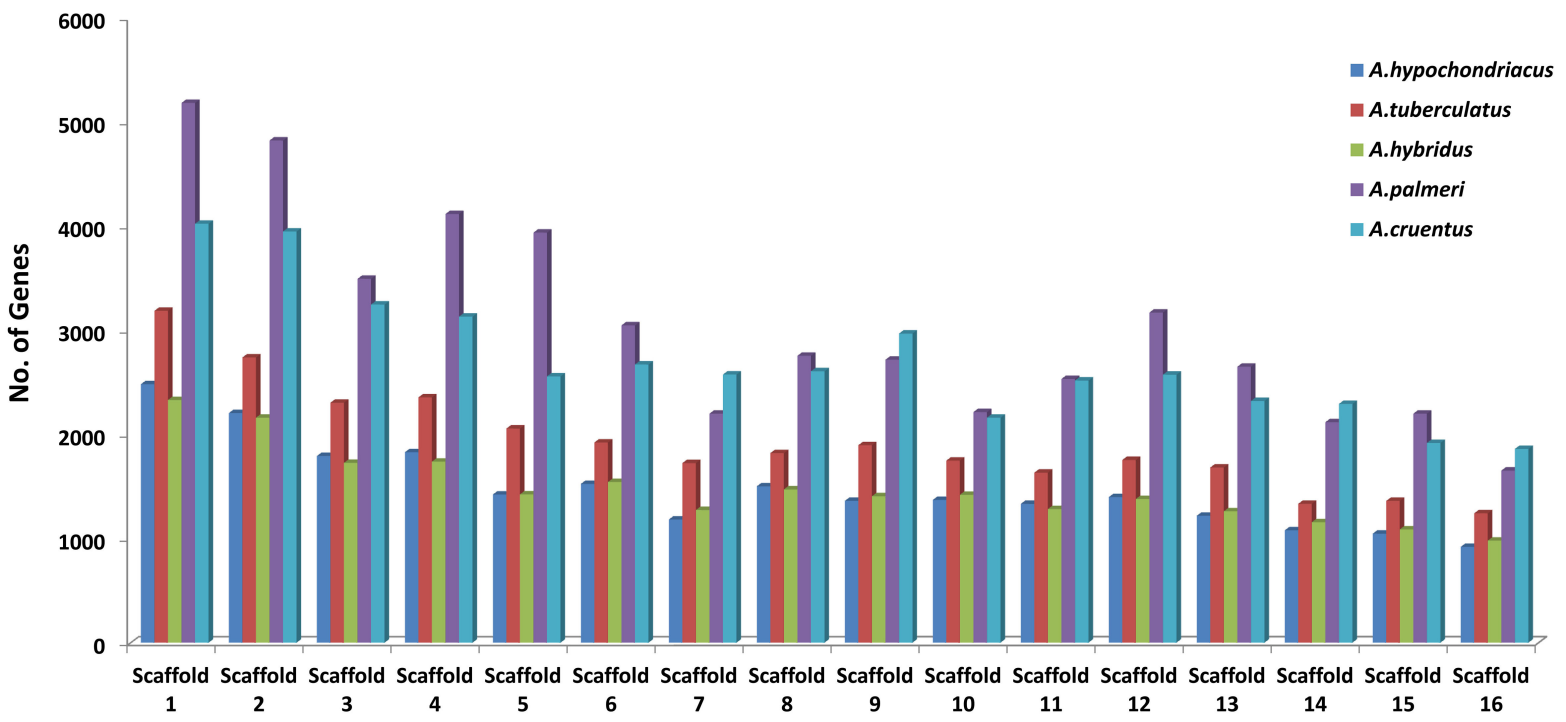
Distribution of protein-coding genes of all five amaranth species across the 16 scaffolds. The horizontal axis shows the scaffold number, and the vertical axis shows the number of genes present in the respective scaffolds. For *A. cruentus*, the protein-coding genes present in both chromosomes 2A and 2B have been combined and labeled as scaffold 2.

A brief overview of the data stored in the AGRDB database is presented in this section. The distributions of genes along with scaffolds are depicted in all five amaranth species (**Figure 6**). The highest number of genes were identified in *A. palmeri* (48,625), followed by *A. cruentus* (43,382), *A. tuberculatus* (30,771), *A. hypochondriacus* (23,879), and *A. hybridus* (23,820), respectively. Scaffold 1 contains the maximum no. of genes, approximately (9.2%–10.4%) of the total genes and scaffold 16 possesses the least number of genes, which covers approximately (3.4%–4.2%) of the total genes (**Figure 6**). Out of the total genes, *A. hypochondriacus* (18,432), *A. tuberculatus* (24,213), *A. hybridus* (19,602), *A. palmeri* (37,245), and *A. cruentus* (32,423) genes have been successfully annotated. Among the predicted SSRs, the maximum number of SSR markers was identified in *A. hypochondriacus* (205,567), followed by *A. tuberculatus* (165,246), *A. palmeri* (106,586), *A. hybridus* (102,492), and *A. cruentus* (78,445) (**Supplementary Table S2**). From the total SSRs predicted among five amaranth species, dinucleotides were the most abundant, followed by tri-nucleotide, tetra-nucleotide, penta-nucleotide, and hexa-nucleotide repeats except in *A. hybridus*, *A. palmeri*, and *A. cruentus*, which had greater numbers of hexa-nucleotide than penta-nucleotide repeats (**Supplementary Table S2**).

**Figure 7:**
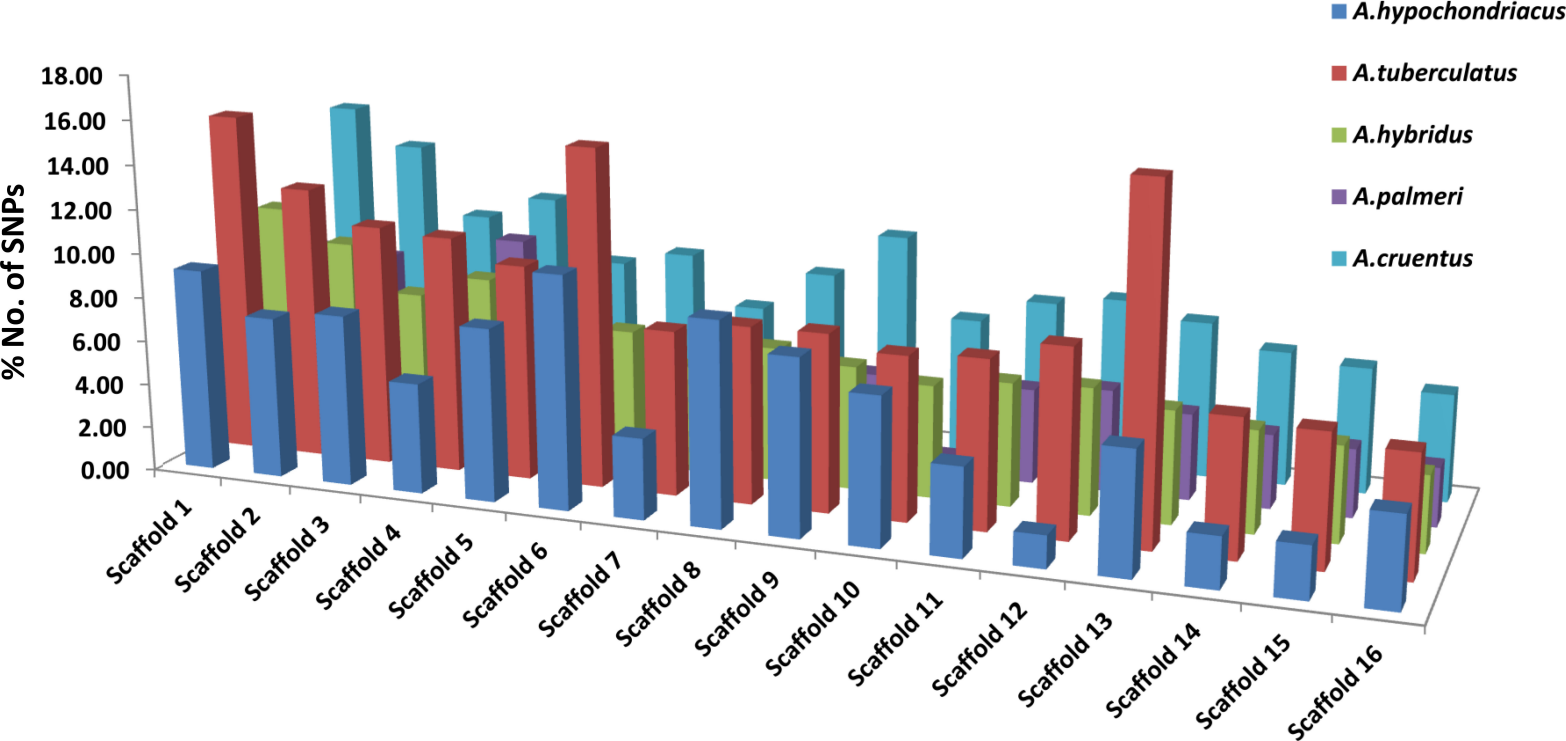
Distribution of SNPs across all 16 scaffolds of *A. hypochondriacus*, *A. tuberculatus*, *A. hybridus*, *A. cruentus*, and *A. palmeri*. The *x*-axis shows the scaffold numbers, and the *y*-axis shows the % No. of SNPs present in each scaffold.

**Figure 8:**
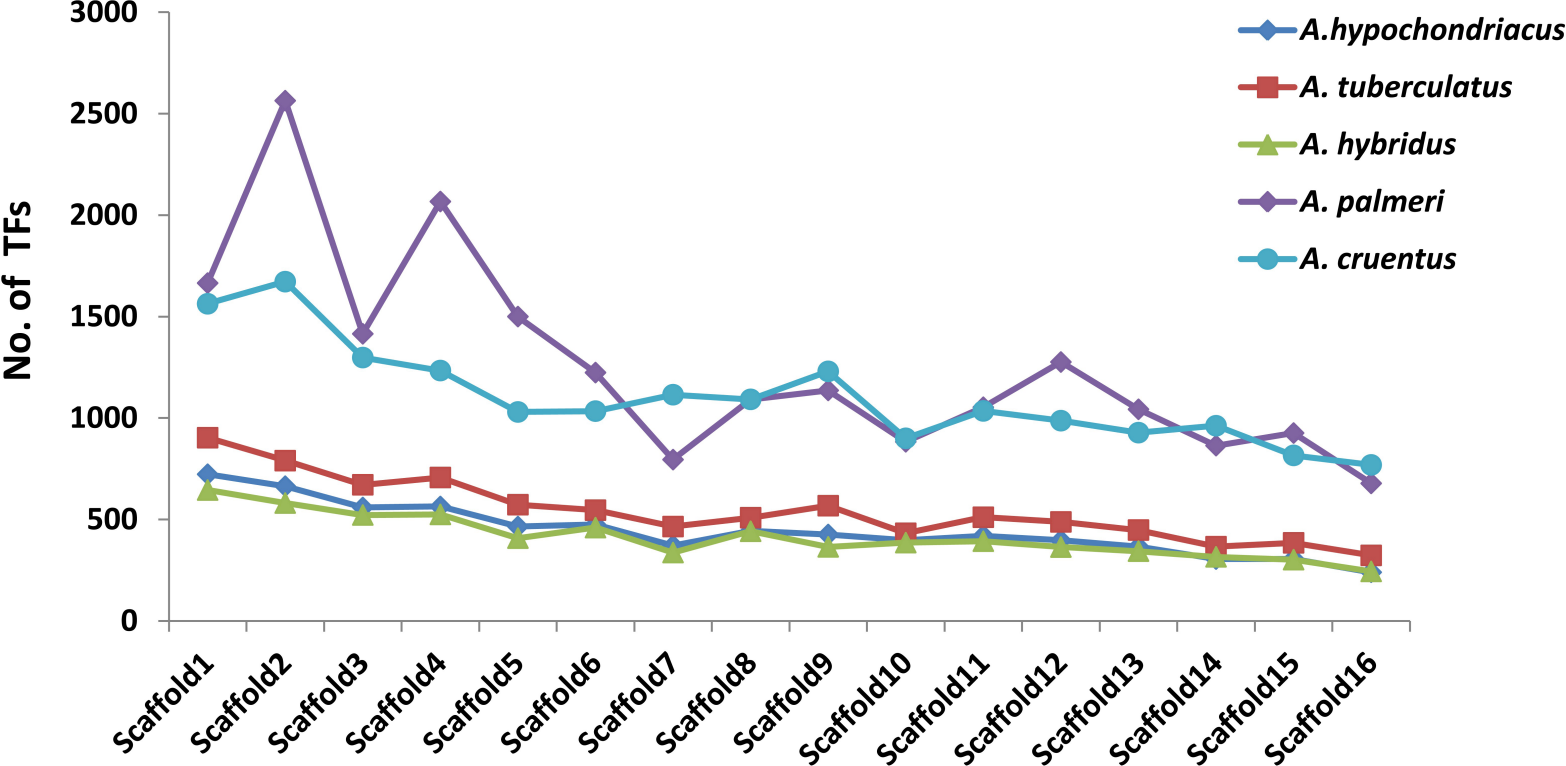
Distribution of TFs across 16 scaffolds of all five amaranth species. The bar line of each species is presented in a different color. The *x*-axis shows the number of scaffolds, and the *y*-axis shows the number of transcription factors. For *A. cruentus*, TFs present in both chromosomes 2A and 2B have been combined, and labeled as scaffold 2.

Among all five amaranth species, *A. hypochondracus*, *A. tuberculatus*, *A.hybridus*, *A. palmeri*, and *A.cruentus*, the SNPs were distributed asymmetrically across 16 scaffolds. In *A. hypochondriacus*, scaffold 6 contains the highest number of SNPs, 106,528 (11.11%), followed by scaffold 8 (9.71%), scaffold 1 (9.70%), scaffold 9 (8.37%), scaffold 5 (8.26%), scaffold 3 (8.17%), and scaffold 12 (1.54%) (**Figure 7**). In *A. tuberculatus*, scaffold 13 contains the highest number of SNPs, 160,174 (10.19%), followed by scaffold 1 (10%), scaffold 6 (9.88%), scaffold 2 (8.01%), scaffold 3 (7.06%), scaffold 4 (6.93%), and scaffold 16 (3.52%) (**Figure 7**). In *A. hybridus*, scaffold 1 contains the highest number of SNPs, 106,720 (10.76%), followed by scaffold 2 (9.31%), scaffold 4 (8.2%), scaffold 3 (7.14%), and scaffold 5 (6.71%). Similarly, in *A. cruentus*, scaffold 1 contains the highest number of SNPs, 142,684 (10.79%), followed by scaffold 2 (9.51%), scaffold 4 (7.9%), scaffold 9 (7.5%), and scaffold 3 (7.0%). While in *A. palmeri*, scaffold 4 contains the highest number of SNPs, 91,732 (12.06%), followed by scaffold 1 (10.54%), scaffold 2 (8.97%), scaffold 5 (7.73%), scaffold 6 (6.58%), scaffold 3 (6.57%), and scaffold 7, which contains the least number of SNPs, 16,358 (approximately 2.15%) of the total SNPs (**Figure 7**). Based on the distribution pattern of TFs across scaffolds, the highest numbers of TFs were present in scaffold 1, followed by scaffold 2, scaffold 4, scaffold 3, and the lowest numbers of TFs were present in scaffold 16 in *A. hypochondriacus*, *A. tuberculatus*, and *A. hybridus* species (**Figure 8**). In *A. palmeri*, the highest numbers of TFs were present in scaffold 2, followed by scaffold 4, scaffold 1, scaffold 5, and the lowest numbers of TFs were present in scaffold 7 (**Figure 8**). While in *A. cruentus*, scaffold 2 had the most TFs, followed by scaffold 1, scaffold 3, scaffold 4, and scaffold 16, which had the least TFs (**Figure 8**).

**Figure 9:**
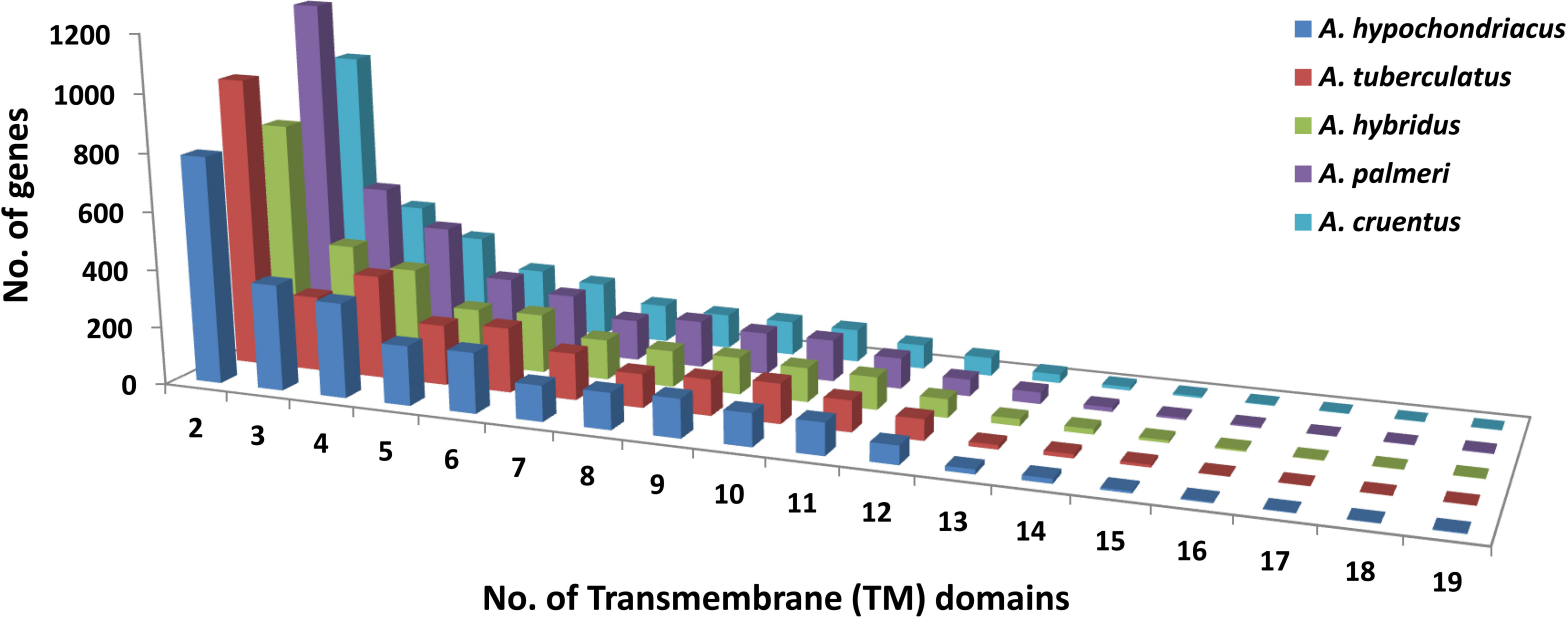
Distribution of transmembrane (TM) domains across the peptide sequences of five Amaranth species, where the *x*-axis shows the number of trans-membrane domains, and the *y*-axis shows the number of genes.

Based on the homology search, a total of 154 pre-miRNAs encoding 42 mature miRNAs belong to 27 miRNA families in *A. hypochondriacus*;126 pre-miRNAs encoding 36 mature miRNAs belonging to 26 miRNA families in *A. tuberculatus*; 121 pre-miRNAs encoding mature miRNAs belongs to 26 miRNA families in *A. hybridus*; 110 pre-miRNAs encoding 38 mature miRNAs belongs to 22 miRNA families in *A. palmeri*; and 119 pre-miRNAs encoding 36 mature miRNAs belonging to 25 miRNA families in *A. cruentus* were predicted (**Supplementary Table S3**). In the case of transporter identification, the highest number of TM proteins was predicted in *A. palmeri* (6,984), followed by *A. tuberculatus* (6,443), *A. cruentus* (5,789), *A. hybridus* (5,106), and *A. hypochondriacus* (5,789) genomes. Out of the total predicted TM proteins, the non-redundant transporter genes carrying two or more transmembrane domains from *A. hypochondriacus* (2,760), *A. tuberculatus* (2,905), *A. hybridus* (2,642), *A. palmeri* (3,402), and *A. cruentus* (2,760) were selected in our analysis (**Figure 9**).

## Discussion

Recent advancements in next-generation sequencing technologies have enabled the sequence, assembly, and annotation of many plant genomes in a very short time and provided genetic information on plant growth, development, and evolution. Genome sequencing and analysis technologies have not only improved our understanding of plant species but also accelerated functional genomics studies and molecular breeding. The databases developed based on the above genomic information can greatly facilitate research related to growth and development, genetics and breeding, and secondary metabolite synthesis pathways in plants. Recently, a range of databases have been developed dedicated to genomic data focusing on specific crop species. For example, the Cucurbit Genomics Database (CuGenDB) comprises all available genome and expressed sequence tag (EST) sequences, genetic maps, and transcriptome profiles for cucurbit species, as well as sequence annotations, biochemical pathways, and results of comparative genomic analysis such as synteny blocks and homologous gene pairs between different cucurbit species (Zheng et al., 2018). The Citrus Genome Database (CGD) is a resource to enable basic, translational, and applied research in citrus. It contains genomics, genetics, and breeding data for citrus species. It also contains 63 citrus genetic maps, 42,238 citrus markers, 479 citrus QTLs, and PathwayCyc for *C. clementina* and *C. sinensis*. (https://www.citrusgenomedb.org/). The *Lonicera japonica* functional genomics database (LjaFGD) includes a *Lonicera japonica* genome and 77 transcriptome profiles, different tools for gene functional analysis, motif analysis, network analysis, and sequence analysis (Xiao et al., 2021). *Catharanthus roseus* functional genomics database (croFGD) contains scaffolded genome sequence of *C. roseus*, 53 RNA-seq datasets of different tissues (flower, root, leaf, seedling, and shoot) with different treatments (MeJA, PnWB infection, and yeast elicitor). It also contains miRNA-target pairs, predicted PPI pairs into the network and provided several tools such as gene set enrichment analysis, functional module enrichment analysis, and motif analysis for functional prediction of the co-expression genes (She et al., 2019). Sorghum Functional Genomics Database (SorghumFDB) is an integrated knowledge base of sorghum gene family classifications (transcription regulators/factors, carbohydrate-active enzymes, protein kinases, ubiquitins, cytochrome P450, monolignol biosynthesis-related enzymes, R-genes, and organelle genes), detailed gene annotations, miRNA and target gene information (Tian et al., 2016). *Setaria italica* Functional Genomics Database (SIFGD) has been established for the bioinformatics analyses of gene function or regulatory modules. It also contains a genome browser to integrate *S. italica* genome sequences, transcript sequences, protein sequences, expressed sequence tags (EST), miRNA-seq, and RNA-seq data which was collected from different data sources (You et al., 2015).

Various studies have reported the development and use of molecular markers such as SSRs (Suresh S. et al., 2014; Delgado and Martín, 2022; Vats et al., 2023), SNPs (Jamalluddin et al., 2022; Hoshikawa et al., 2023); for the diversity analysis but their application in marker-assisted improvement of grain amaranth is very limited. The availability of high-density SNPs information for various amaranth species makes them valuable for genome mapping, analyzing genetic diversity and population structure, constructing ultra-high-density genetic maps, map-based positional cloning, and providing genotypes for genome-wide association analysis (Xia et al., 2019). Over the last two decades, SSRs and SNPs have become the most popular molecular markers and are potentially used for studying the population’s genetic structure and reconstructing the evolutionary history of species (Tsykun et al., 2017). Transcription factors and miRNAs are master regulators of the plant cellular system. Only a few studies are reported about genome-wide gene family identification (Hajyzadeh, 2023) and miRNA identification (Martínez Núñez et al., 2021). Further exploration or validation of the important genes related to plant development and defense response can be done by using the AGRDB database as a base. The majority of miRNA targets are TFs, which are very crucial for regulating plant growth and development. Different TF families which regulate a variety of regulatory networks may help plants to grow in both normal as well as stressed environmental conditions (Samad et al., 2017). Similarly, transporters are also an important class of membrane proteins that facilitate the exchange of solutes including diverse molecules and ions across the cellular membrane, which are very essential for the survival of any organism (Deshmukh et al., 2020), which can be explored further in amaranth as well as other related crop species using database information.

The AGRDB database developed in the present study provides users with a comprehensive genomic resource for amaranth species. The AGRDB database contains a variety of datasets, which includes 170,477 functionally annotated protein-coding genes, 658,336 SSRs, 5,602,117 SNPs, 59,649 TFs, 191 miRNAs, and 14,469 transporters generated from the extensive analysis of five *Amaranthaceae* species. The functional annotation of the genes allowed us to classify them into different functional classes, which can be a very useful resource in determining the physiological significance of a large number of genes. A comparison of the AGRDB database with similar types of existing databases based on different features shows the present database fulfills, all the features mentioned in other databases (**Table 3**).

**Table 3:** A comparison of the AGRDB database with other related databases.

Features	AGRDB	TGRD	TeaPGDB	CuGenDB
Number of species	5	1	7	16
Gene details	Yes	Yes	Yes	Yes
Functional annotation of genes	Yes	Yes	Yes	Yes
Microsatellites (SSRs)	Yes	Yes	Yes	Yes
SSR primer details	Yes	Yes	Yes	Yes
Genic and nongenic classification of SSRs	Yes	Yes	Yes	Yes
Single nucleotide polymorphism (SNP)	Yes	Yes	Yes	Yes
Transcription factors (TFs)	Yes	Yes	No	Yes
Putative miRNAs	Yes	Yes	No	Yes
miRNA-targeted genes	Yes	Yes	No	Yes
Transporters	Yes	No	No	No
BLAST search	Yes	Yes	Yes	Yes
Genome browser	Yes	No	Yes	Yes
Synteny viewer	No	No	No	Yes
Gene expression	Yes	Yes	No	Yes

The AGRDB database has provisions for updating. The transcriptomic, metabolomic, proteomic, phenomic, and ionomics data will also be incorporated into the AGRDB database based on the availability of the data. Whenever high-quality genome sequence data of new *Amaranth* species will be available, the data of respective species will be analyzed and added to the AGRDB database, which can also be used to perform comparative genomic analyses. In the future, more extensive features such as plotting functions, gene co-expression network analysis and comparative genome analysis platform may also be included in the database to meet the demands of amaranth researchers as well as to make it more user-friendly.

## Conclusion and future developments

The AGRDB database is a comprehensive genomic resource for the genus *Amaranthus*, including the species *A. hypochondriacus*, *A. tuberculatus*, *A. hybridus*, *A. palmeri*, and *A. cruentus*. The AGRDB database, which is freely available and accessible via the web address http://www.nbpgr.ernet.in:8080/AmaranthGRD/, contains genomic information such as genes, SSRs, SNPs, TFs, miRNAs, and transporters of five amaranth species at one place.

## Data availability statement

The datasets presented in this study can be found in online repositories. The names of the repository/repositories and accession number(s) can be found in the article/**Supplementary Material**.

## Author contributions

RS conceived and designed the experiments. AS performed data curation and formal data analysis and developed the database resource. AKM and AM performed formal data analysis. AS performed visualization, software, and written original draft of the manuscript. RS supervised the study and provides funding acquisition. SR, AKS, RB, SKK, SK, VG, KS, and RS writing—review and editing the manuscript. All authors contributed to the article and approved the submitted version.
