# Supplementary material for: Amaranth Genomic Resource Database: an integrated database resource of Amaranth genes and genomics
Source: Front Plant Sci. 2023 Jun 28;14:1203855. doi: 10.3389/fpls.2023.1203855 (PMC10337998; doi:10.3389/fpls.2023.1203855)
Supplement: Supplementary file 1 [file DataSheet_1.docx]

Supplementary Material

Amaranth Genomic Resource Database (AGRDB): an integrated database resource of Amaranth genes and genomics

Akshay Singh^1^, Ajay Kumar Mahato^2^, Avantika Maurya^1^, S Rajkumar^1^, A. K. Singh^1^, Rakesh Bhardwaj^1^, S. K. Kaushik^1^, Sandeep Kumar^1^, Veena Gupta^1^, Kuldeep Singh^3^ and Rakesh Singh ^1,*^

* Correspondence: Rakesh Singh; rakesh.singh2@icar.gov.in

# Supplementary Figures

**Supplementary Figure 1.** miRNA prediction pipeline used for genome-wide insilico putative miRNA identification from five amaranth species.

**
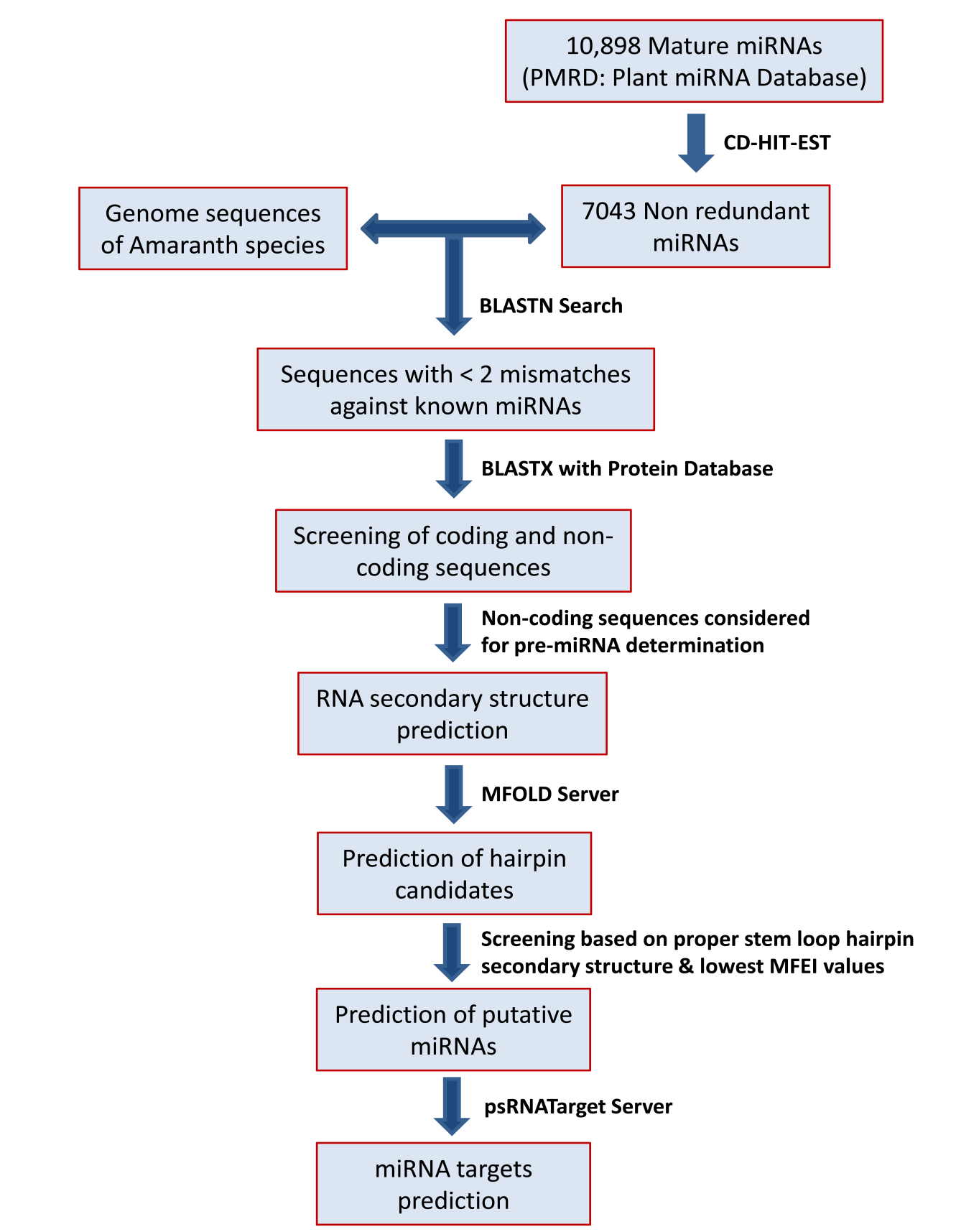
**

**Supplementary Figure 2.** Description of the schema architecture used to construct AGRDB database.

**
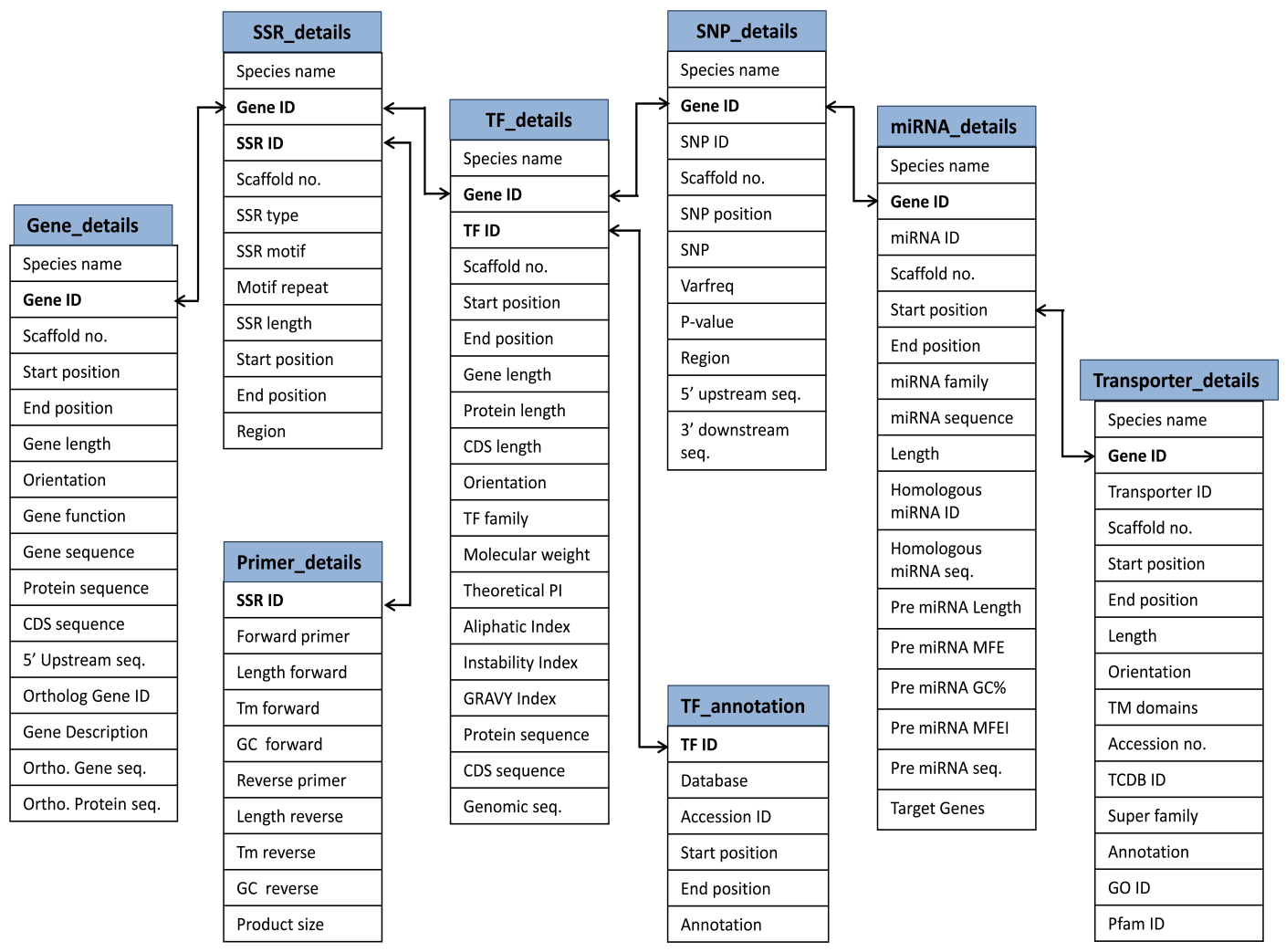
**

**Supplementary Figure 3.** Showing Gene Expression search module with the output bar chart for queried gene based on RPKM values.

**
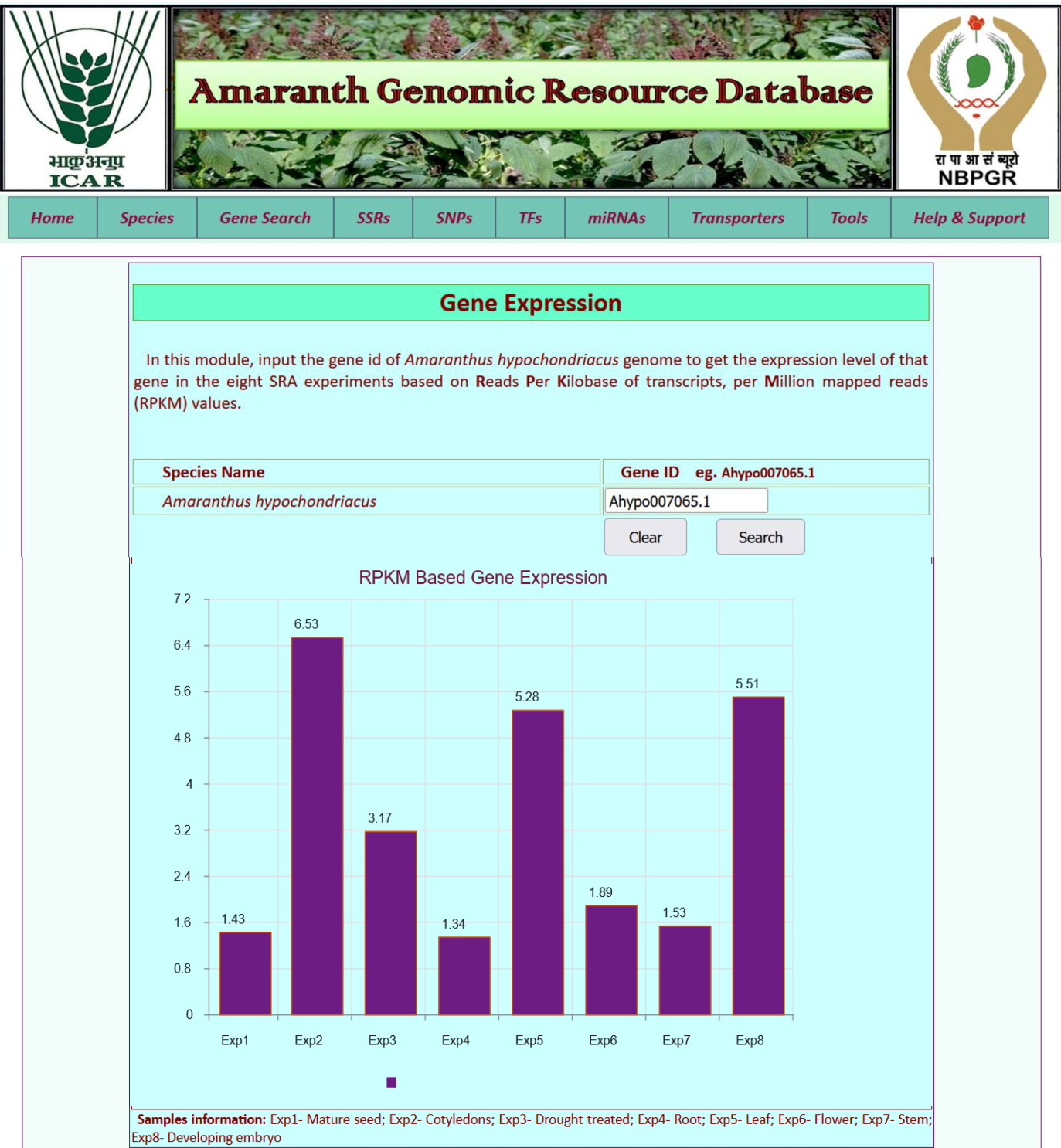
**

**Supplementary Figure 4.** Showing local BLAST Search page with the available nucleotide as well as protein databases.


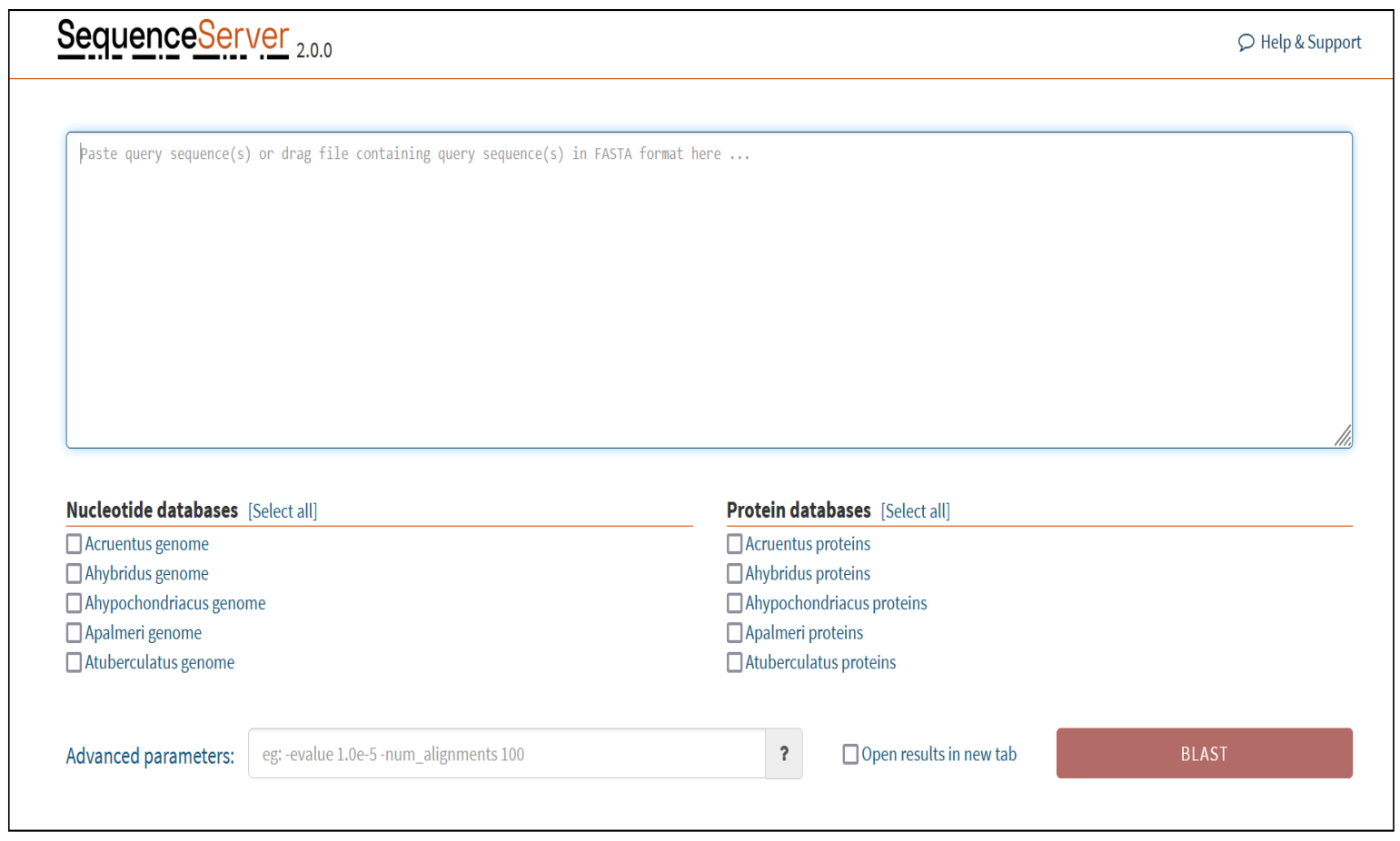


**Supplementary Figure 5.** Showing the JBrowse genome browser with the available tracks, such as Genes, SNPs, SSRs, and TFs along with reference genome sequence.

##
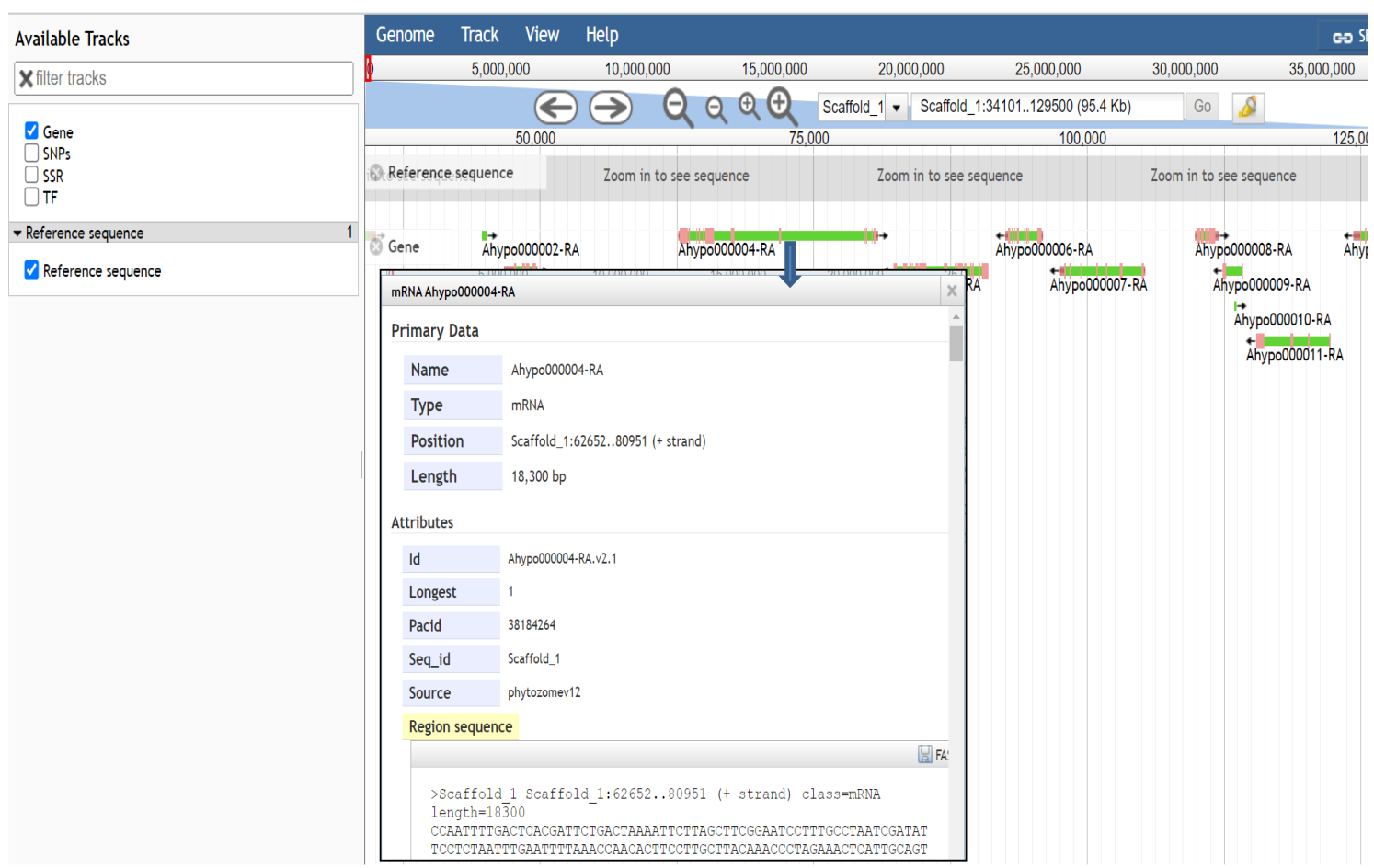


## Supplementary Tables

**Supplementary Table 1.** List of BioProjects and no. of SRA accessions used for the genome wide SNPs identification in five amaranth species.

| **Species** | **BioProject ID** | **No. of SRA Samples** |
| --- | --- | --- |
| ***A. hypochondriacus*** | PRJNA290898 | 8 |
| ***A. tuberculatus*** | PRJNA432348 | 48 |
| ***A. palmeri*** | PRJNA626536 | 18 |

**Supplementary Table 2.** Distribution of predicted SSR motif (di- to hexa-) nucleotide types across all five amaranth species.

| **Species** | **Total SSRs** | **Di** | **Tri** | **Tetra** | **Penta** | **Hexa** |
| --- | --- | --- | --- | --- | --- | --- |
| ***A. hypochondriacus*** | 205,567 | 154,264 | 39,351 | 6,851 | 2,691 | 2,410 |
| ***A. tuberculatus*** | 165,246 | 96,683 | 62,166 | 3,227 | 1,977 | 1,193 |
| ***A. hybridus*** | 102,492 | 67,127 | 31,152 | 2,572 | 605 | 1,036 |
| ***A. palmeri*** | 106,586 | 69,482 | 33,118 | 2,241 | 775 | 970 |
| ***A. cruentus*** | 78,445 | 50,555 | 24,792 | 2,018 | 454 | 626 |

**Supplementary Table 3.** Putative miRNAs and their target genes identified in five amaranth species by genome wide analysis.

| **Species** | **No. of pre-miRNAs** | **Mature miRNAs** | **miRNA families** | **No. of targets** |
| --- | --- | --- | --- | --- |
| ***A. hypochondriacus*** | 154 | 42 | 27 | 512 |
| ***A. tuberculatus*** | 126 | 36 | 26 | 869 |
| ***A. hybridus*** | 121 | 39 | 26 | 707 |
| ***A. palmeri*** | 110 | 38 | 22 | 658 |
| ***A. cruentus*** | 119 | 36 | 25 | 365 |
